# Integrated xenosurveillance of *Loa loa*, *Wuchereria bancrofti*, *Mansonella perstans* and *Plasmodium falciparum* using mosquito carcasses and faeces: A pilot study in Cameroon

**DOI:** 10.1371/journal.pntd.0010868

**Published:** 2022-11-02

**Authors:** Joseph Pryce, Nils Pilotte, Benjamin Menze, Allison R. Sirois, Michael Zulch, Jean Pierre Agbor, Steven A. Williams, Charles S. Wondji, Lisa Reimer

**Affiliations:** 1 Department of Vector Biology, Liverpool School of Tropical Medicine, Liverpool, United Kingdom; 2 Department of Biological Sciences, Smith College, Northampton, Massachusetts, United States of America; 3 Molecular and Cellular Biology Program, University of Massachusetts, Amherst, Massachusetts, United States of America; 4 Centre for Research of Infectious Diseases, Yaoundé, Cameroon; Task Force for Global Health, UNITED STATES

## Abstract

**Background:**

Community presence of loiasis must be determined before mass drug administration programmes for lymphatic filariasis and onchocerciasis can be implemented. However, taking human blood samples for loiasis surveillance is invasive and operationally challenging. A xenosurveillance approach based on the molecular screening of mosquitoes and their excreta/feces (E/F) for *Loa loa* DNA may provide a non-invasive method for detecting the community presence of loiasis.

**Methods:**

We collected 770 wild mosquitoes during a pilot study in a known loiasis transmission area in Mbalmayo, Cameroon. Of these, 376 were preserved immediately while 394 were kept in pools to collect 36-hour E/F samples before processing. Carcasses and E/F were screened for *L*. *loa* DNA. To demonstrate this method’s potential for integrated disease surveillance, the samples were further tested for *Wuchereria bancrofti*, *Mansonella perstans*, and *Plasmodium falciparum*.

**Results:**

Despite limited sample numbers, *L*. *loa* DNA was detected in eight immediately-stored mosquitoes (2.13%; 95% CI 1.08 to 4.14), one carcass stored after providing E/F (0.25%; 95% CI 0.04 to 1.42), and three E/F samples (estimated prevalence 0.77%; 95% CI 0.15 to 2.23%). *M*. *perstans* and *P*. *falciparum* DNA were also detected in carcasses and E/F samples, while *W*. *bancrofti* DNA was detected in E/F. None of the carcasses positive for filarial worm DNA came from pools that provided a positive E/F sample, supporting the theory that, in incompetent vectors, ingested parasites undergo a rapid, complete expulsion in E/F.

**Conclusions:**

Mosquito xenosurveillance may provide a useful tool for the surveillance of loiasis alongside other parasitic diseases.

## Author summary

Loiasis is a typically-mild filarial worm disease. In contrast, lymphatic filariasis (LF) and onchocerciasis are diseases of significant public health concern. LF and onchocerciasis are primarily controlled by administering chemotherapeutics to entire at-risk human populations. However, individuals with heavy loiasis infections are at risk of severe adverse events when treated with these drugs. Accurate loiasis surveillance is therefore critical to the safe implementation of these programmes.

Screening humans to detect parasites in the blood is invasive and expensive at large scale. Recently, however, several studies have demonstrated the potential of collecting wild blood-feeding insects such as mosquitoes and screening them for the presence of human blood-borne parasites. To explore the potential of mosquito screening as a non-invasive tool for loiasis surveillance, we collected mosquitoes in a known loiasis transmission area in Cameroon. We tested the samples for parasites that cause loiasis, and concurrently screened for the parasites that cause malaria, LF and mansonellosis to determine the potential for integrated disease surveillance. The DNA of all four parasites were identified using these methods. The findings suggest mosquito screening may provide a useful tool for the surveillance of loiasis alongside other parasitic diseases.

## Background

Loiasis is a filarial disease caused by *Loa loa* parasites, vectored primarily by flies of the genus *Chrysops*. The distribution of this disease is limited to central Africa, with 80% of the 14 million people at risk of loiasis living in Cameroon or the Democratic Republic of Congo [1]. The symptoms of loiasis are typically benign, and historically it has not been considered a disease of significant health importance [2–4]. However, the presence of loiasis has become a cause for concern in recent years as it poses a serious operational challenge to the elimination programmes for both lymphatic filariasis (LF) and onchocerciasis [5]. When treated with diethylcarbamazine (DEC) or ivermectin, drugs that are integral to the mass drug administration (MDA) programmes used to control onchocerciasis and LF, individuals with heavy *L*. *loa* worm burdens are at risk of severe neurological reactions, encephalopathy, and death [6]. In areas that are potentially endemic for loiasis, the presence or absence of the infection must therefore be determined before mass treatment regimens can be utilised [7]. Consequently, a precise way of identifying areas that are endemic for loiasis is a priority for both onchocerciasis control programmes and the Global Programme to Eliminate Lymphatic Filariasis (GPELF).

Several methods have been used to map loiasis distribution. Epidemiological surveys, in which blood samples are collected from entire populations and screened for the presence of *L*. *loa* microfilariae, provide a direct indication of the current community prevalence. Recently, these approaches have benefited from the development of field-friendly diagnostics such as the LoaScope–a simple optical device that can be coupled to a smartphone to count *L*. *loa* mf in peripheral blood without the need for sample processing [8]. However, these methods remain naturally invasive, and the blood-screening of entire populations can be operationally challenging at large scale [1]. Instead, the African Programme for Onchocerciasis Control (APOC) currently recommends the use of Rapid Assessment Procedure for Loiasis (RAPLOA) surveys, in which local populations are questioned about the history of loiasis symptoms in their community [9]. A history of subconjunctival larval presence is a strong predictor of high community prevalence [1]. However, in many areas surveyed by RAPLOA, the results are unclear and further data are required before a decision can be made on whether or not to implement MDA [1]. Given the RAPLOA’s focus on the history of symptoms in a surveyed area, results also have limited scope to provide an assessment of current endemicity.

Molecular xenomonitoring (MX), the detection of pathogen DNA in a vector species, can be used as a proxy for the presence of a pathogen in the human population and provides a non-invasive and relatively inexpensive alternative to epidemiological surveys. MX methods have been increasingly adopted by elimination programmes for filarial diseases and are recommended by both the GPELF and APOC for the surveillance of LF and onchocerciasis, but have not been used for the surveillance of loiasis [10–12]. A key challenge is the lack of effective standardized methods for the collection of adult *Chrysops* flies. The most commonly used method is to equip local community members with hand nets to catch the flies as they look for a bloodmeal, a laborious process that is heavily dependent on individual ability and attention [10].

Xenosurveillance—the molecular screening of blood-feeding insects for ingested blood-borne pathogens that they may not necessarily act as vectors for—has the potential to contribute to loiasis mapping efforts as part of an integrated disease surveillance programme. Mosquitoes provide a convenient sampling target for xenosurveillance due to their relative abundance and because many endemic areas have existing infrastructure for mosquito collections due to their role in malaria transmission. Recent studies have also demonstrated the potential of screening mosquito excreta/feces (E/F) for the genetic material of pathogens, providing a non-destructive sampling approach with improved scope for pooling [13,14]. Xenosurveillance of mosquitoes and their E/F has shown strong potential for the community-level detection of multiple pathogens including blood-borne viruses [15,16], malaria parasites [17,18] and the filarial pathogens *Wuchereria bancrofti*, *Brugia malayi*, and *Mansonella perstans* [17,18]. As *L*. *loa* microfilariae have been detected in night blood of infected individuals, *L*. *loa* parasite DNA may also be taken up in sufficient quantities by night-biting mosquitoes to be detected through xenosurveillance methods [19,20].

The primary objective of this study was to determine whether *L*. *loa* DNA can be recovered from mosquitoes and their E/F in loiasis-endemic communities. A secondary objective was to demonstrate the potential of integrated xenosurveillance for multiple diseases by concurrently screening collected samples for the filarial worms *W*. *bancrofti* and *M*. *perstans*, and the malaria parasite *Plasmodium falciparum*. To guide future xenosurveillance sampling strategies, a final objective was to report the different prevalence estimates given when measured using a variety of methods.

## Methods

### Study area

**Fig 1:**
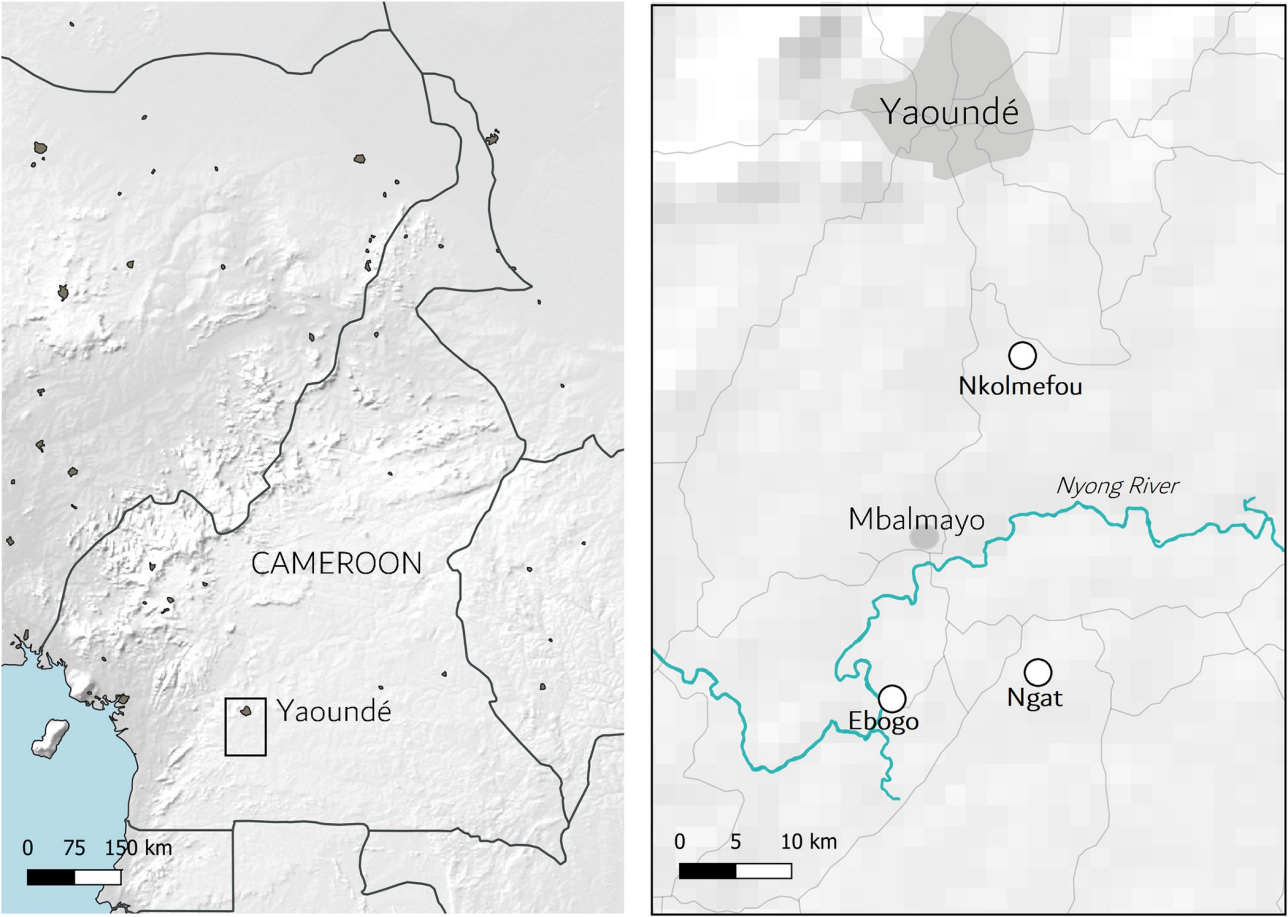
Map of the study area, showing the location of the Nyong river basin and the three villages in which sampling was conducted: Nkolmefou, Ngat, and Ebogo. Made with Natural Earth. Free vector and raster map data @ naturalearthdata.com.

The study was conducted in June 2019 in the Mbalmayo district in the Centre Region of Cameroon. The town of Mbalmayo is positioned approximately 50 km south of the capital city of Yaoundé in the river basin of the Nyong River, where the surrounding swamps and forested environment provide ideal breeding sites for *Chrysops* flies (Fig 1). The mean annual temperature is 24.1°C and the mean annual rainfall is 1600 mm [7]. The district has a history of high loiasis prevalence, with a prevalence of 27.3% reported earlier in 2019 [7,21,22]. Sampling was conducted in three villages in the district—Ngat, Ebogo and Nkolmefou–chosen based on the observed presence of *Chrysops* flies. During a preliminary survey in the sampling area, two species of *Chrysops* (*Ch*. s*ilacea* and *Ch*. *dimidiata*) were collected and dissected, and the presence of infectious L3 *L*. *loa* larvae was confirmed. The study objectives and proposed strategy were discussed and agreed upon with each village Chief prior to conducting the survey.

### Sample collection

Indoor resting mosquitoes were collected from each consenting household between 0600 and 1000 hours using battery-powered aspirators. Collections were repeated until every household in each village had been visited and invited to participate at least once. In total, collections were conducted over nine days, with four, three, and two days spent in Nkolmefou, Ngat, and Ebogo, respectively. BG-sentinel 2 traps (Biogents, Germany) equipped with BG-sweetscent artificial lures were utilised concurrently to indoor resting collections. Four traps, positioned a minimum of 50 m apart, were set at 1800 hours on the evening prior to the indoor resting collection and collected at 1700 hours the following evening. Any collected male mosquitoes were discarded.

*Chrysops* fly collections were conducted on the same day and in the same village as mosquito collections. *Chrysops* flies were collected by two trained fly collectors equipped with hand nets and asked to collect host-seeking flies between 1000 and 1600 hours. To provide a standardized measure of *Chrysops* fly abundance, two Nzi traps were also set in each village for one day, between 1000 and 1600 hours [23]. All collected *Chrysops* flies were considered to be host-seeking and therefore assumed to be female.

### E/F collection and mosquito storage

Half of the collected female mosquitoes were killed and stored following collection. Prior to storage, the species and abdomen status (unfed, blood-fed or gravid) of each mosquito were identified using a dissecting microscope. Carcasses were then stored individually in 1.5 ml microcentrifuge tubes containing silica gel.

The remaining mosquitoes were stored alive for 36 hours to collect E/F using the methods described by Cook *et al* [13]. Briefly, a paper cone was constructed which was coated with NeverWet (Rust-Oleum, Durham, UK) to create a superhydrophobic surface, and a DNA collecting card (GenTegra) was placed beneath the opening at the bottom of the cone. Mosquitoes of any genera or species collected from the same household were pooled together in a single cone. The mosquito-containing cones were stored in a cool box with the lid open and partially covered with a damp towel to keep the mosquitoes cool and humid. Cotton wool soaked in a 10% sugar solution was added to the netting covering each cone. After 36 hours, the cones were dismantled and the cards were stored in 1.5 ml microcentrifuge tubes. In the event of E/F deposits drying directly onto the cones, the cones were stored and later swabbed for DNA extraction following the swabbing methods described in Cook *et al*. [13]. Due to a shortage of cones, ten of the E/F samples were collected directly onto paper cups. The whole cups were stored, and the E/F samples were later swabbed for DNA extraction. Mosquitoes from which E/F had been collected were then knocked down for species identification and stored individually as described above.

### DNA extraction

Molecular processing of E/F samples was conducted at Smith College, Northampton, MA, USA, while processing of mosquito and fly carcasses was conducted at the Liverpool School of Tropical Medicine, UK. DNA was extracted from E/F samples using the methods previously described by Pilotte *et al* [18]. Briefly, a standard paper punch was used to take three punches from each collection card, and sample was eluted/recovered from these punches using the GenSolve DNA Recovery Kit (GenTegra). DNA was then extracted from each sample using the MinElute column procedure (Qiagen, Germantown, MD). Extraction of DNA from mosquito and fly carcasses was conducted using DNeasy extraction kits (Qiagen) with a mechanical disruption stage using a TissueLyser bead mill (Qiagen) as previously described [17].

### Sample analysis using qPCR

All mosquito and E/F samples were tested for the presence of *W*. *bancrofti*, *P*. *falciparum*, *L*. *loa*, and *M*. *perstans* using species-specific qPCR assays. *Chrysops* samples were tested for the presence of *L*. *loa*. Presence/absence determinations occurred using 2.5 μL of template DNA and previously described assays in accordance with the published protocols [24,25]. All samples were tested in duplicate using each assay, and samples resulting in amplification from both replicates were scored as “positive”. Samples which failed to produce a positive result for either replicate reaction were scored as “negative”. If duplicate testing results were discordant, the sample was tested a second time, again in duplicate reactions. If either or both of the re-test reactions resulted in an amplification product, the sample was scored as a “positive” and the re-test results were reported. If both re-test replicates failed to produce a product, a negative result was reported for the sample.

### W. bancrofti confirmatory testing using an LDR qPCR assay

The presence of a significant number of *W*. *bancrofti*-positive E/F samples, coupled with an absence of *W*. *bancrofti*-positive mosquito carcass samples represented an unexpected finding. As a result, qPCR-based confirmatory testing was performed. This testing occurred using a qPCR assay based on detection of a long DNA repeat of *W*. *bancrofti* (LDR; GenBank accession no. AY297458) [26]. This assay was chosen because it targets a region of the *W*. *bancrofti* genome that is entirely independent from the TR1 target region utilized by the primary assay [25]. Such targeting controlled for plasmid control-based or PCR product-based sample contamination. All samples that tested positive for *W*. *bancrofti* with the TR1 qPCR assay were retested using this LDR assay. All samples were tested in duplicate and run in accordance with published conditions using 5 μL of template. In the event of insufficient sample volume, residual volumes were split between replicates and sterile water was added to increase template volume to 5 μL for addition to reaction wells.

### Analysis

For each parasite, we calculated the prevalence of parasite DNA in mosquito carcasses and associated 95% confidence intervals using the E. B. Wilson method and software available at http://vassarstats.net/prop1.html [27]. We calculated separate prevalence estimates for mosquitoes that were stored immediately and those that were stored after providing a 36-hour E/F sample.

For E/F samples collected from pools of mosquitoes, we considered the pool to have produced a positive E/F sample if the GenTegra card and/or the swabbed cone or cup were found to be positive for parasite DNA. We calculated maximum likelihood estimates of the prevalence of parasite DNA in the mosquito population using PoolScreen v2 [28]. We explored the correlation between parasites detected in pooled E/F samples with the carcasses of individual mosquitoes that contributed to them.

We further reported the difference in prevalence estimates observed between different mosquito genera, bloodfed status and methods of collection (indoor resting collection vs. BG-Sentinel 2 trap).

## Results

### Sample collection

In total, 770 female mosquitoes were collected during the study. Of these, 376 mosquitoes were stored immediately following collection, including 297 *Anopheles*, 53 *Culex*, 17 *Aedes* and 9 *Mansonia* species mosquitoes. The remaining 394 mosquitoes, comprising 386 *Anopheles* spp. mosquitoes and 8 *Culex* spp. mosquitoes were kept for 36 hours to collect E/F. No *Aedes* or *Mansonia* species were deemed fit for E/F collection, as all those collected had died or become injured during the capture process. In total, E/F samples were collected from 59 mosquito pools, from 52 distinct households, with a median pool size of 3 (range 1 to 38). Additionally, 312 *Chrysops* flies were collected, with one fly collected in an Nzi trap and all other flies caught manually. Both *Ch*. *silacea* (n = 192) and *Ch*. *dimidiata* (n = 120) were present.

### Detection of L. loa DNA

A summary of the detection rates of each of the four parasites in each of the three mosquito sample types is provided in Table 1. In total, nine mosquito carcasses were positive for *L*. *loa* DNA. Of the 376 mosquitoes stored immediately after collection, eight were positive for *L*. *loa* DNA (2.13%; 95% CI 1.08 to 4.14), with five of these collected from a single household. The remaining three were collected from three different households, with one in each of the three villages. In contrast, just one of the 394 carcasses stored after providing a 36-hour E/F sample were positive (0.25%; 95% CI 0.04 to 1.42). Three of the 59 collected mosquito E/F samples were also found to contain *L*. *loa* DNA, corresponding to a maximum likelihood estimated prevalence of *L*. *loa* DNA in collected mosquitoes of 0.77% (0.15 to 2.23%). The three positive E/F samples were collected from three different households, across both villages in which E/F surveys took place. Of the 312 Chrysops flies collected in the study area, 108 were found to be positive for *L*. *loa* DNA (34.62%, 95% CI 29.56 to 40.06%).

**Table 1:** DNA detection rates of L. Loa; W. bancrofti; M. perstans and P. falciparum in the carcasses of mosquitoes stored immediately, the carcasses of mosquitoes stored after a 36-hour E/F collection window, and in collected E/F samples.

		*Loa loa*		*Wuchereria bancrofti*		*Mansonella perstans*		*Plasmodium falciparum*	
Sample type	No. screened	No. Positive	Prevalence % (95% CI)	No. Positive	Prevalence % (95% CI)	No. Positive	Prevalence % (95% CI)	No. Positive	Prevalence % (95% CI)
Mosquito E/F pools^ⴕ^	59	3	0.77 (0.15 to 2.23)	10^‡^	2.94 (1.32 to 5.50)	4	1.09 (0.28 to 2.80)	21	8.37 (4.87 to 13.28)
Mosquito carcass (post-E/F)	394	1	0.25 (0.04 to 1.42)	0	0.00 (0.00 to 0.97)	2	0.51 (0.14 to 1.84)	145	36.80 (32.19 to 41.67)
Mosquito carcass (immediate storage)	376	8	2.13 (1.08 to 4.14)	0	0.00 (0.00 to 1.01)	0	0.00 (0.00 to 1.01)	212	56.38 (51.33 to 61.30)

Footnotes

^ⴕ^ Prevalence calculated using PoolScreen v2.0

^‡^ Using TR1 qPCR assay

### Detection of W. bancrofti, M. perstans and P. falciparum DNA

No *W*. *bancrofti* DNA was detected in any of the carcasses. However, ten E/F samples were found to contain *W*. *bancrofti* DNA using the TR1 target (estimated prevalence 2.94%; 95% CI 1.32 to 5.50%). Upon confirmatory qPCR testing using the LDR target, four of the ten E/F samples were confirmed to contain *W*. *bancrofti* DNA in both replicates, while two further samples were positive for *W*. *bancrofti* DNA in one of two replicates.

Although none of the immediately-stored mosquito carcasses were positive for *M*. *perstans* DNA (95% CI 0.00 to 1.01), two post-E/F carcasses (0.51%; 95% CI 0.14 to 1.84) and four E/F samples (estimated prevalence 1.09%; 95% CI 0.28 to 2.80%) were positive.

In addition, 212 immediately-stored mosquito carcasses (56.38; 95% CI 51.33 to 61.30), 145 post-E/F carcasses (36.80%; 95% CI 32.19 to 41.67) and 21 E/F samples (estimated prevalence 8.37%; 95% CI 4.87 to 13.28%) were positive for *P*. *falciparum* DNA.

### Correlation between E/F samples and corresponding post-E/F carcasses

**Fig 2:**
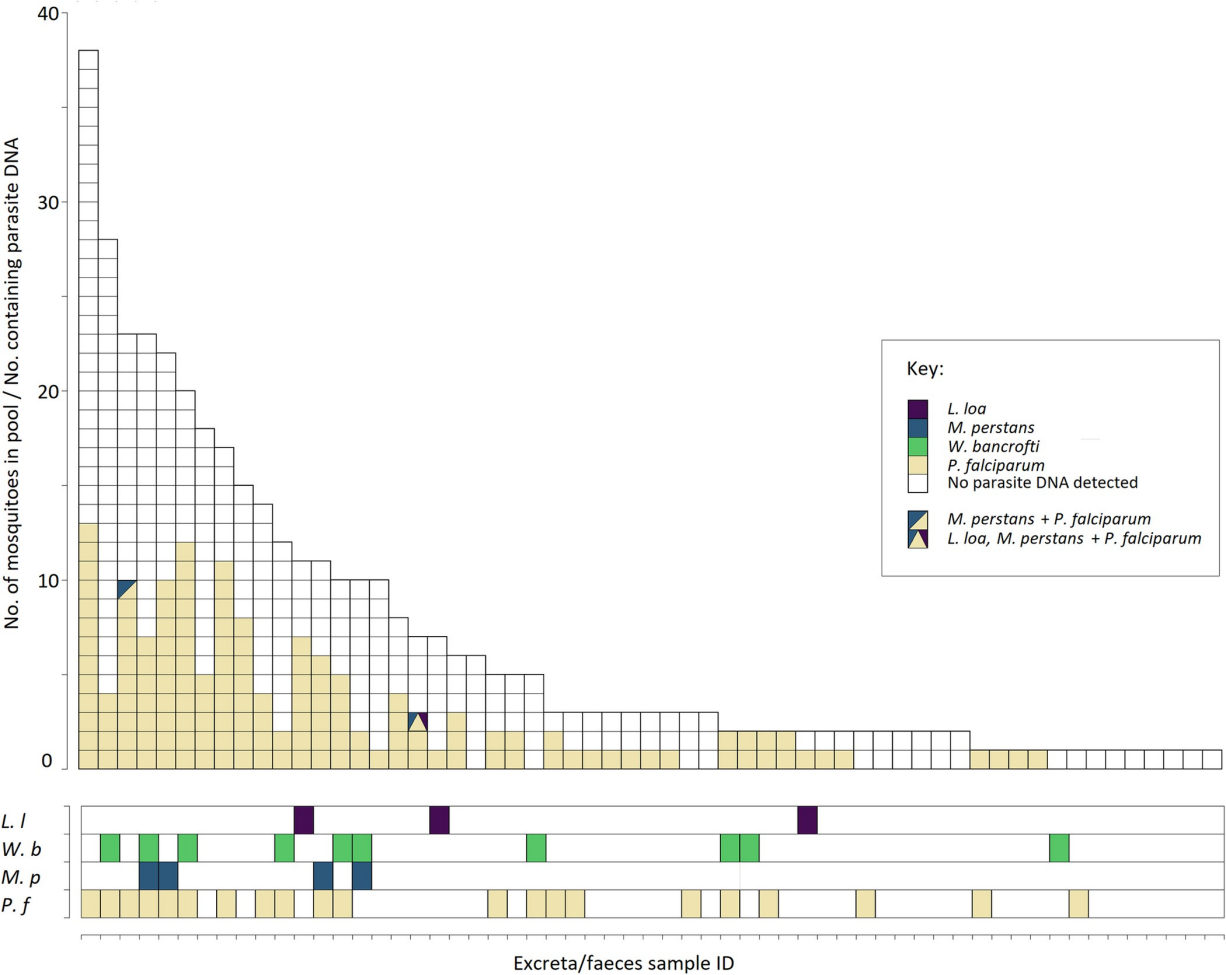
Upper: The number of mosquitoes contributing to each of the 59 excreta/feces samples and proportion of individual mosquitoes found to be positive for *L*. *loa*, *M*. *perstans*, *W*. *bancrofti* or *P*. *falciparum* DNA. Lower: corresponding presence or absence of the four parasites’ DNA in each of the collected excreta/feces samples. Abbreviations: L. l–*L*. *loa*; W. b–*W*. *bancrofti*; M. p–*M*. *perstans*; P. f–*P*. *falciparum*.

Fig 2 compares the detection of parasite DNA in collected E/F samples with the corresponding detection in the carcasses of mosquitoes that contributed to each sample. Notably, none of the carcasses positive for filarial worm DNA had been housed in any of the cones that provided a positive E/F sample.

### Comparison between collection methods, mosquito genera and blood-fed status

In this study, only 31 mosquitoes were collected using BG-Sentinel traps, none of which were in adequate condition to allow for the collection of an E/F sample. No mosquito carcasses collected using BG-Sentinel traps were positive for any parasite. Each of the carcasses and excreta samples identified as positive for at least one parasite were therefore obtained using indoor resting collections.

All the mosquito carcasses positive for *L*. *loa* or *M*. *perstans* DNA were *Anopheles* spp. mosquitoes and all were blood-fed. In contrast, *P*. *falciparum* DNA was detected in the carcasses of *Anopheles* (49.34%, 337/683), *Aedes* (41.18%, 7/17), *Culex* (9.84%, 6/61) and *Mansonia* spp. mosquitoes (77.78%, 7/9). Although the highest detection rates of *P*. *falciparum* were observed in blood-fed mosquitoes (75.41%, 138/183), the parasite was also detected in gravid (46.15%, 30/65) and unfed mosquito carcasses (20.63%, 13/63).

Of the ten E/F samples positive for *W*. *bancrofti*, nine came exclusively from pools of *Anopheles* mosquitoes and one sample came from a single *Culex* mosquito. All four E/F samples positive for *M*. *perstans* came from pools of *Anopheles* mosquitoes, while the single *L*. *loa* positive E/F sample came from a pool containing six *Anopheles* and one *Culex* mosquito.

## Discussion

Given the limitations of existing surveillance tools for the detection of areas with cases of loiasis, the primary objective of this study was to identify whether or not MX methods can be used to detect *L*. *loa* DNA in mosquitoes and their E/F. In an area of known loiasis transmission, we detected both mosquito carcasses and E/F samples positive for *L*. *loa* DNA, giving cause for optimism that mosquitoes can provide a suitable sampling target for identifying loiasis-positive communities.

The prevalence of *L*. *loa* DNA was significantly higher in *Chrysops* flies than mosquitoes. Potential explanations for this finding lie in the larger blood-meals taken by *Chrysops* flies [29,30] and the diurnal periodicity of *L*. *loa* corresponding with the peak biting activity of *Chrysops* flies [31]. As the overwhelming majority of mosquitoes collected in this study were night-biting *Anopheles* species, a greater mosquito prevalence of *L*. *loa* DNA may be observed in areas where day-biting mosquitoes are predominant, though future work will be needed to evaluate this. Nevertheless, despite the high proportion of night-biting species and limited collection effort, carcasses and E/F positive for *L*. *loa* DNA were detected in each village in which surveys were conducted. This suggests the sensitivity of mosquito xenosurveillance was suitable for the purposes of identifying loiasis-positive communities. Mosquitoes may therefore provide a convenient alternative sampling target to *Chrysops* flies, particularly as Nzi traps were found to be ineffective in this study and collection of *Chrysops* depended entirely on human collectors.

Furthermore, mosquito-based xenosurveillance may be more sustainable than traditional MX of *Chrysops* flies due to its potential for integrated surveillance with existing malaria programmes and other neglected tropical diseases. To further demonstrate this potential, we concurrently screened collected mosquitoes and E/F samples for *W*. *bancrofti*, *M*. *perstans* and *P*. *falciparum*, successfully identifying all three parasites. The detection of *W*. *bancrofti* DNA in E/F samples represented an unexpected finding, as a recent lymphatic filariasis mapping exercise by Wanji and colleagues found no evidence of *W*. *bancrofti* endemicity in Cameroon [32]. As the detection of *W*. *bancrofti* was confirmed using a second PCR assay with a distinct target DNA sequence, the potential for this finding to be a result of contamination is highly unlikely. A limitation of the methods utilised in this study is that they do not distinguish between mosquitoes that have recently ingested a bloodmeal containing parasite DNA, and those with a mature infection that are capable of transmitting the disease. Therefore, the detection of *W*. *bancrofti* DNA in mosquito E/F alone is not indicative of LF transmission. However, the study area was not included in the recent survey by Wanji *et al*. [32]. Further investigation may therefore be required to confirm the absence of transmission.

To guide future sampling strategies for mosquito-based surveillance of loiasis and other parasitic diseases, a final objective was to observe the difference in detection rates when using a variety of sample types and collection methods. Typically, filarial worm detection rates were lower in the carcasses of mosquitoes that had been stored following the provision of a 36-hour E/F sample than in the E/F samples themselves and the carcasses of mosquitoes that were stored immediately after capture. In addition, positive E/F samples were not associated with positive post-E/F mosquito carcasses. Taken together, these findings support the theory that, in incompetent vectors, ingested parasites undergo a rapid and complete expulsion in E/F, after which the parasite is no longer detectable in the carcass [18]. The reduced detection in mosquito carcasses processed 36 hours after collection highlights the critical importance to xenosurveillance activities of an appropriate sampling strategy and timely processing of samples. Were a surveillance programme to store collected mosquitoes in cages for this time period prior to processing, without the collection of E/F, the sensitivity to each parasite may be dramatically reduced. This importance is further exemplified by the absence of parasite detection in any trap-caught mosquitoes, which would likely have undergone longer delays between taking a blood-meal and storage than those caught resting indoors.

Traditional epidemiological surveys based on the collection of human blood samples provide information about infection status at an individual patient level. In contrast, xenosurveillance, and questionnaire-based approaches such as RAPLOA, provide information at a community level. A challenge to such approaches lies in the possibility of communities that have low overall rates of infection but which contain individuals with high microfilarial burdens and thus have a greater risk of suffering severe adverse events. Further research will be necessary to determine whether the presence or absence of *L*. *loa* DNA in mosquitoes can provide sufficient information to make a decision on whether or not to implement MDA.

### Conclusions

In loiasis-endemic communities, *L*. *loa* DNA can be recovered from night-biting mosquitoes and their E/F. Mosquito xenosurveillance may provide a useful tool for the real-time integrated surveillance of loiasis presence alongside malaria and other filarial worm diseases. Due to the transient nature of parasite DNA signals in the carcasses and E/F of non-vector mosquitoes, the use of appropriate sampling methods that minimise the delay between blood-feeding and processing of samples is essential.
